# Comment on “Extreme Oncoplastic Surgery for Multifocal/Multicentric and Locally Advanced Breast Cancer”

**DOI:** 10.1155/2019/4693794

**Published:** 2019-06-02

**Authors:** Gianluca Franceschini, Alba Di Leone, Riccardo Masetti

**Affiliations:** Division of Breast Surgery, Department of Women's and Children's Health, Fondazione Policlinico Universitario Agostino Gemelli IRCCS, Università Cattolica del Sacro Cuore, Largo A. Gemelli, 8-00168 Rome, Italy

We read with interest the work of Chaitanyanand Koppiker and colleagues [1] and would like to add some useful suggestions to improve oncological and cosmetic outcomes.

Extreme oncoplastic surgery (EOS) is a breast conserving operation, using oncoplastic techniques, in a patient who requires a mastectomy. It is a promising new concept in the multidisciplinary treatment of selected patients with generally large, greater than 5 cm multifocal or multicentric tumor desiring breast conservation [2–4].

These oncoplastic techniques allow the resection of a greater amount of breast tissue with safer margins and acceptable aesthetic results increasing breast conservation rates [5–8].

The indications for EOS are different and various algorithms have been devised to assist with the decision process [3–5]. Long-term data on recurrence and survival are not available, using this approach. It is expected that the local recurrence will be somewhat higher but that there will be little or no impact on survival [3, 9].

Chaitanyanand Koppiker and colleagues conclude in their study that “Extreme Oncoplastic Surgery followed by RT results in acceptable local-regional control, low rate of complications, and high patient satisfaction. In selected patients, EO could provide a safe alternative for breast conservation surgery instead of mastectomy” [1].

However, a “great surgical hand” in EOS is not enough to optimize the oncological and aesthetic results! We think that a good oncoplastic surgery requires both individual ability and technical skill but also other attributes as dedication, decision-making skills, and the repetitive performance of specific tasks.

So, while performing the extreme oncoplastic breast conserving surgery, the modern oncoplastic surgeon should always follow some specific and crucial steps, such as careful local staging of the disease with ultrasonography, mammography, and magnetic resonance before surgery; adequate radiological preoperative study with localization of tumor and/or calcifications; multidisciplinary discussion, in a dedicated “surgery board”, to choose an oncoplastic technique tailored to patient; intraoperative ultrasound to guide the resection; intraoperative radiological and pathological evaluation of the specimen for definition of lesion and margins of resection; frozen sections should be obtained from a portion of all six faces of the resected specimen; systematic circumferential tumor cavity shaving to have a backup to lumpectomy margins; placement of clips within the excision cavity as a “landmark” to define the tumor bed and guide adjuvant breast radiotherapy; accurate pathological assessment of the specimen using macrosections.

This multidisciplinary path leads more easily to tumor-free margins by keeping the amount of healthy breast tissue excision as low as possible; while performing oncoplastic procedures, it would be useful to bear in mind these recommendations and suggestions in order to improve oncological and cosmetic outcomes. The modern breast surgeon must always know where they are going and the repetitive performance of specific tasks could enhance their ability when faced with the extreme and complex oncoplastic procedures.
